# Supplementary material for: Correlation measurement of propagating microwave photons at millikelvin
Source: Nat Commun. 2025 Apr 24;16:3875. doi: 10.1038/s41467-025-59230-2 (PMC12022262; doi:10.1038/s41467-025-59230-2)
Supplement: Supplementary file 1 — Supplementary Information [file 41467_2025_59230_MOESM1_ESM.pdf]

Supplemental Information for
“Correlation measurement of propagating microwave photons at millikelvin”

(Dated: March 31, 2025)

CONTENTS

Supplementary Note 1. Unit of propagating photons 1

Supplementary Note 2. Response of electron temperature 2

Supplementary Note 3. Broadening of reflection spectrum 2

Supplementary Note 4. Experimental setup 3

Supplementary Note 5. Detailed fitting procedure 5

Supplementary Note 1. UNIT OF PROPAGATING PHOTONS

Photons are excitations of electromagnetic fields that may have multiple degrees of freedom. The characteristics of photons depend on the modes that are used to describe the field. In the case of cavity quantum electrodynamics, it is convenient to define the modes as standing waves confined in the cavity volume. The corresponding Hamiltonian operator, $\hbar\omega_k \hat{a}_k^\dagger \hat{a}_k$, yields the energy of excitation of the kth mode, where ω_k is its angular frequency. The discrete field operators obey the commutation relation $\left[\hat{a}_{k_1}, \hat{a}_{k_2}^\dagger\right] = \delta_{k_1,k_2}$. However, this treatment encounters challenges in describing a stationary beam of radiation where energy is not a conserved quantity. Indeed, it is more convenient to describe a propagating field with continuous modes [1], after which a single-mode approximation may be carried out to simplify the discussion.

We define the instantaneous field operator at the input of the nanobolometer as $\hat{a}(t)$ with the commutation relation $\left[\hat{a}(t_1), \hat{a}^\dagger(t_2)\right] = \delta(t_1 - t_2)$. Its Fourier transform is given by

$$\hat{a}(\omega) = \frac{1}{\sqrt{2\pi}} \int_{-\infty}^{\infty} \hat{a}(t) \mathrm{e}^{\mathrm{i}\omega t} \, \mathrm{d}t, \tag{1}$$

and obeying the commutation relation $\left[\hat{a}(\omega_1), \hat{a}^\dagger(\omega_2)\right] = \delta(\omega_1 - \omega_2)$. From this definition, we obtain the photon flux operator, i.e., photon number per unit time, as

$$\hat{n}(t) = \hat{a}^\dagger(t)\hat{a}(t) = \frac{1}{2\pi} \int_{-\infty}^{\infty} \int_{-\infty}^{\infty} \hat{a}^\dagger(\omega_1)\hat{a}(\omega_2) \mathrm{e}^{\mathrm{i}(\omega_1 - \omega_2)t} \, \mathrm{d}\omega_1 \mathrm{d}\omega_2. \tag{2}$$

For a stationary beam of radiation, $\langle \hat{n}(t) \rangle$ is independent of the specific time index t. Therefore, we may write [1]

$$\hat{a}^\dagger(\omega_1)\hat{a}(\omega_2) = 2\pi\hat{n}(\omega_1)\delta(\omega_1 - \omega_2). \tag{3}$$

Here, the factor of 2π is introduced for obtaining the compact result $\hat{n}(t) = \int_{-\infty}^{+\infty} \mathrm{d}\omega \, \hat{n}(\omega)$. This choice indicates that $\hat{n}(\omega)$ is the photon flux density per angular frequency.

For a spectrally filtered field, we define $\hat{a}_f(\omega) = f^*(\omega)\hat{a}(\omega)$. Here, $f(\omega)$ is the spectral profile of the filter with $\int_{-\infty}^{+\infty} |f(\omega)|^2 \mathrm{d}\omega = \mathcal{N}$ where $\mathcal{N}$ is a normalization factor. We have [2]

$$\hat{a}_f(t) = \frac{1}{\sqrt{2\pi}} \int_{-\infty}^{+\infty} f^*(\omega)\hat{a}(\omega) \mathrm{e}^{-\mathrm{i}\omega t} \, \mathrm{d}\omega. \tag{4}$$

One can check that $[\hat{a}_f(t), \hat{a}_f^\dagger(t)] = 1$ if we define $\mathcal{N} = 2\pi$. Equivalently, we can also write

$$\hat{a}_f(t) = \frac{1}{\sqrt{2\pi}} \int_{-\infty}^{+\infty} f^*(t - t')\hat{a}(t') \, \mathrm{d}t', \tag{5}$$

where $f(t)$ is the Fourier transform of $f(\omega)$. This equation indicates that, at each time instance t, the field that passes through the filter is a weighted average of the input field at all times.

Let us define the power operator of a stationary beam of radiation as

$$\hat{P}_\mathrm{h}(t) = \int_{-\infty}^{\infty} \hbar\omega\hat{n}(\omega) \, \mathrm{d}\omega. \tag{6}$$

Our goal is to build a relation between the power and the operator $\hat{a}_f(t)$ for propagating fields. In the simplest case, we define [2]

$$f(\omega) = \begin{cases} 1/\sqrt{\mathrm{FWHM}}, & \text{for } |\omega - \omega_0|/(2\pi) < \mathrm{FWHM}/2; \\ 0, & \text{elsewhere.} \end{cases} \tag{7}$$

Here, FWHM is the bandwidth of the filter with central frequency $\omega_0/(2\pi)$. We may rewrite the instant power as

$$\hat{P}_\mathrm{h}(t) = \mathrm{FWHM} \times \int_{-\infty}^{\infty} |f(\omega)|^2 \hbar\omega\hat{n}(\omega) \, \mathrm{d}\omega. \tag{8}$$

We therefore obtain $\hat{P}_\mathrm{h}(t) \approx \mathrm{FWHM} \times \hbar\omega_0 \hat{a}_f^\dagger(t)\hat{a}_f(t)$ if FWHM is small compared to the central frequency $\omega_0/(2\pi)$. Thus, we obtain

$$\langle \hat{a}_f^\dagger(t)\hat{a}_f(t)\rangle \approx \frac{\langle \hat{P}_\mathrm{h}\rangle}{\mathrm{FWHM}\hbar\omega_0}. \tag{9}$$

This is the relation for determining the photon number defined in the unit of photons/(s × Hz). For thermal radiation fields at temperature T, we have [3]

$$\langle \hat{a}_f^\dagger(t)\hat{a}_f(t)\rangle = \frac{1}{\exp\left[(\hbar\omega_0)/(k_\mathrm{B}T)\right] - 1}. \tag{10}$$

This is one formalism of the celebrated Planck's law.

Supplementary Note 2. RESPONSE OF ELECTRON TEMPERATURE

At the nanobolometer, the thermodynamics of the electron temperature is given by

$$C_\mathrm{e}\dot{T}_\mathrm{e} = \hat{P}_\mathrm{h} - \Sigma V\left(T_\mathrm{e}^{n+1} - T_\mathrm{b}^{n+1}\right), \tag{11}$$

where C_e is the electron heat capacity, T_e the electron temperature, T_b the phonon temperature, and ΣV the product of material- and geometry-dependent constants. Here, $\hat{P}_\mathrm{h}$ the instantaneous power absorbed by the nanobolometer, which we treat as a statistical variable owing to quantum fluctuations.

We consider temperature fluctuations around the equilibrium point $\langle \hat{P}_\mathrm{h}\rangle = \Sigma V\left(T_\mathrm{e}^{n+1} - T_\mathrm{b}^{n+1}\right)$. The equilibrium electron temperature assumes the form

$$T_\mathrm{e}^\mathrm{eq} = \left[T_\mathrm{b}^{n+1} + \frac{\mathrm{FWHM}\times\hbar\omega_0}{\Sigma V}\langle \hat{a}_f^\dagger(t)\hat{a}_f(t)\rangle\right]^{1/(n+1)}. \tag{12}$$

The temperature change around T_e^eq integrated over the response time of the nanobolometer Δt is a statistical variable, which may be described as

$$\delta\hat{T}_\mathrm{e} = \frac{1}{C_\mathrm{e}}\int_t^{t+\Delta t}\delta\hat{P}_\mathrm{h}\,\mathrm{d}t\,, \tag{13}$$

where $\delta\hat{P}_\mathrm{h}(t) = \mathrm{FWHM}\times\hbar\omega_0\left[\hat{a}_f^\dagger(t)\hat{a}_f(t) - \langle\hat{a}_f^\dagger(t)\hat{a}_f(t)\rangle\right]$. In our case, where the integration time is much longer than the average time interval between two adjacent photon emission events, i.e., $\Delta t \gg 1/(2\pi\times\mathrm{FWHM})$, the central limit theorem indicates that the temperature fluctuations described by $\delta\hat{T}_\mathrm{e}$ can be treated as Gaussian with zero mean.

Next, we calculate explicitly the variance of $\delta\hat{T}_\mathrm{e}$. Because $\langle\delta\hat{T}_\mathrm{e}\rangle = 0$, the variance of δT_e is equivalent to

$$\begin{aligned}\langle(\delta\hat{T}_\mathrm{e})^2\rangle &= \left(\frac{\mathrm{FWHM}\times\hbar\omega_0}{C_\mathrm{e}}\right)^2\int_t^{t+\Delta t}\int_t^{t+\Delta t}\left\langle\left[\hat{a}_f^\dagger(t_1)\hat{a}_f(t_1) - \langle\hat{a}_f^\dagger(t_1)\hat{a}_f(t_1)\rangle\right]\left[\hat{a}_f^\dagger(t_2)\hat{a}_f(t_2) - \langle\hat{a}_f^\dagger(t_2)\hat{a}_f(t_2)\rangle\right]\right\rangle\mathrm{d}t_1\mathrm{d}t_2\\ &= \left(\frac{\mathrm{FWHM}\times\hbar\omega_0\Delta n}{C_\mathrm{e}}\right)^2\int_t^{t+\Delta t}\int_t^{t+\Delta t}G\left(t_1,t_2\right)\,\mathrm{d}t_1\mathrm{d}t_2\end{aligned} \tag{14}$$

Here, Δn is square root of the photon number variance, where $(\Delta n)^2 = \langle\left[\hat{a}_f^\dagger(t)\hat{a}_f(t)\right]^2\rangle - \langle\hat{a}_f^\dagger(t)\hat{a}_f(t)\rangle^2$. In addition, $G(t_1,t_2)$ is the two-time correlation of $\hat{a}_f^\dagger(t_1)\hat{a}_f(t_1)$ and $\hat{a}_f^\dagger(t_2)\hat{a}_f(t_2)$. For completely uncorrelated photons, we have $G\left(t_1,t_2\right) = \delta(t_1 - t_2)$ such that $\langle(\delta\hat{T}_\mathrm{e})^2\rangle = \left(\mathrm{FWHM}\times\hbar\omega_0\Delta n/C_\mathrm{e}\right)^2\Delta t$. On the other hand, for perfectly correlated signals, we have $G\left(t_1,t_2\right) = 1$ and therefore $\langle(\delta\hat{T}_\mathrm{e})^2\rangle = \left(\mathrm{FWHM}\times\hbar\omega_0\Delta n/C_\mathrm{e}\right)^2(\Delta t)^2$. We observe that the variance of $\delta\hat{T}_\mathrm{e}$ is proportional to the photon number variance, $(\Delta n)^2$, in both limits.

Supplementary Note 3. BROADENING OF REFLECTION SPECTRUM

The nanobolometer consists of an absorber and a readout resonator, which share the same normal metal nanowire but are electronically isolated by a ground line in the middle (Supplementary Fig. 1). We model the thermometer as a

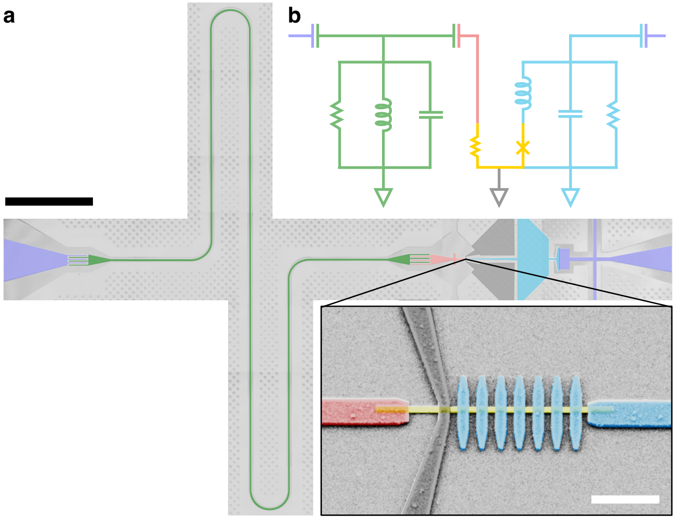

Supplementary Fig. 1: Photograph of sample and equivalent circuit. **a** False colored optical image of a reference sample. The circuit elements are color coded as launch pad (purple), transmission-line filter (green), absorber (red), ground line (gray), and thermometer (blue). The normal-metal nanowire across the absorber and the thermometer is colored in yellow. The scale bar denotes 500 μm. The inset shows a scanning electron microscopy (SEM) micrograph of the long- and short-SNS junctions of the nanobolometer, where the scale bar denotes 2 μm. **b** Equivalent lumped-element circuit of the system with the same color code as in (a).

parallel RLC circuit, described by a resistance R, inductance L, and capacitance C, which is capacitively coupled to the feedline via a small capacitor, C_g. The characteristic impedance of the feedline is assumed to be $Z_0 = 50\,\Omega$. Assuming that $2\pi f_\mathrm{r} Z C_\mathrm{g} \ll 1$ with $Z = \sqrt{L/C}$, we expand the input impedance in a Taylor series around the resonance frequency, f_r, and obtain the input impedance as $Z_\mathrm{in} = R' - i2L'\Delta f$ [4], where $R' = 1/(8\pi Z C_\mathrm{g}^2 Q_\mathrm{i} f_\mathrm{r}^2)$, $L' = 1/(8\pi Z C_\mathrm{g}^2 f_\mathrm{r}^3)$, and Q_i is the internal quality factor of the RLC circuit. The external quality factor is $Q_\mathrm{e} \equiv Q_\mathrm{i} R'/Z_0$. Thus, we obtain the internal and external damping rates as $\gamma_\mathrm{i} = 2\pi f_r/Q_\mathrm{i}$ and $\gamma_\mathrm{c} = 4ZZ_0C_\mathrm{g}^2 f_\mathrm{r}$ [5]. Assuming that Q_i is a constant and f_r varies in a small relative range, we treat γ_i to be independent of f_r whereas γ_c is a fitting parameter for obtaining the reflection coefficient.

The non-averaged reflection coefficient of the thermometer is described by $S_{11} = 1 - \mathrm{e}^{\mathrm{i}\phi}\gamma_\mathrm{c}/\left[(\gamma/2) + \mathrm{i}\Delta\right]$ [5], where $\gamma = \gamma_\mathrm{c} + \gamma_\mathrm{i}$ is the total damping rate and $\Delta = 2\pi(f_\mathrm{r} - f_\mathrm{p})$ is the detuning between the resonance and the probe frequencies. The parameter ϕ describes the asymmetry of the resonance. As discussed in Supplementary Note 2, the temperature fluctuations given by the quantum-statistics of the photons absorbed to the nanobolometer are Gaussian, and hence also the frequency fluctuations of the resonator are accurately described by a Gaussian distribution $\mathcal{N}\left(\mu, \sigma^2\right)$ of mean μ and variance σ^2. Consequently, the temporally averaged reflection coefficient of the nanobolometer probe port is given by averaging the complex-valued non-averaged reflection coefficients over the Gaussian distribution as

$$S_{11}^\mathrm{ave}(f_\mathrm{p}) = \int_{-\infty}^{\infty} \frac{1}{\sqrt{2\pi\sigma^2}} \exp\left[\frac{(f_\mathrm{r}-\mu)^2}{2\sigma^2}\right] S_{11}(f_\mathrm{p}; f_\mathrm{r})\,\mathrm{d}f_\mathrm{r} \tag{15}$$

$$= 1 - \frac{\mathrm{e}^{\mathrm{i}\phi}\gamma_\mathrm{c}}{2\sqrt{2\pi}\sigma}\mathrm{erfcx}\left[\frac{(\gamma/2) + \mathrm{i}2\pi(\mu - f_\mathrm{p})}{2\sqrt{2}\pi\sigma}\right], \tag{16}$$

where erfcx$(\cdot)$ is the scaled complementary error function. The magnitude of S_{11}^ave is equivalent to the well-established Voigt profile encountered, for example, in laser spectroscopy [6].

Supplementary Note 4. EXPERIMENTAL SETUP

The cryogenic and room-temperature setup for the experiment is shown in Supplementary Fig. 2. We use a directional coupler at the thermometer input for reflection-type measurement, where the input signal is attenuated by approximately 20 dB but almost all the signal reflected from the sample is routed to the output port. The signal is subsequently amplified and down converted to an intermediate frequency of 62.5 MHz for measurement. Here, the

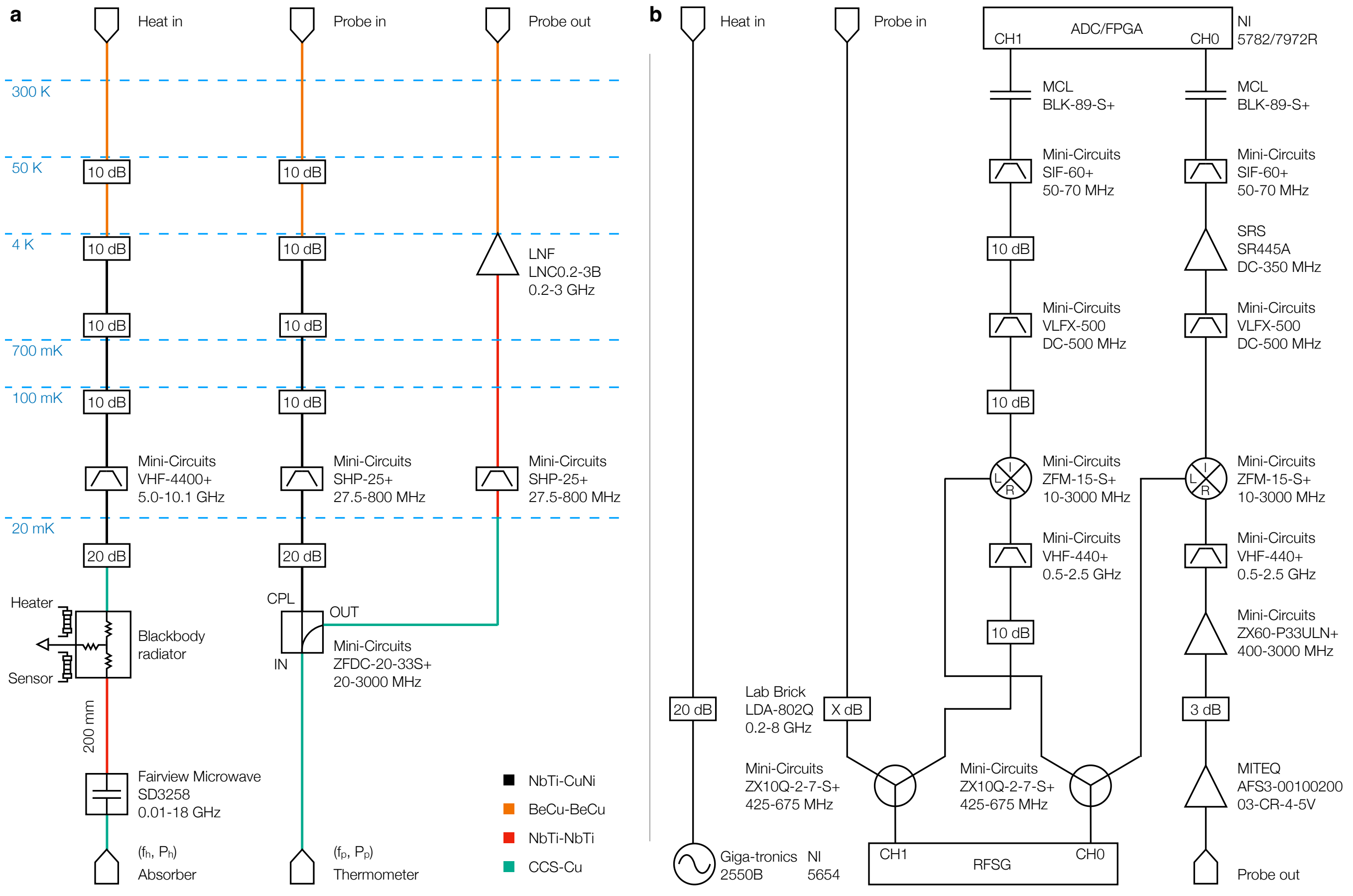

Supplementary Fig. 2: The detailed experimental setup. **a** The sample is mounted at the MXC plate of a Bluefors LD250 dilution refrigerator. Signals are routed between the cryogenic and room temperatures via coaxial microwave cables with different materials along with different microwave components for maximizing the signal-to-noise ratio. The blackbody radiator is suspended below the MXC plate and is weakly thermalized to the latter for local temperature control. **b** The room-temperature setup for photon moments measurement. The probe-out signal is amplified and down-converted for data acquisition, while the probe-in signal is also recorded simultaneously for phase reference.

filters before and after the mixers are used to remove the red sideband. The sampling rate is 250 MS/s. We obtain one data point of the IQ quadratures in each 16 ns period, which is then digitally filtered by a 500 kHz low-pass finite impulse response filter before averaging. The repetition rate of the measurement is 1.25 kHz. In each repetition, we obtain a 32 μs-long trace of the IQ quadratures, which is subsequently averaged by 2×10^4 times.

In our experiment, we use a blackbody radiator to generate thermal photons. Construction of the radiator is based on an 20 dB attenuator as a microwave beam splitter with an $\Gamma = 0.01$ transmission rate of the coherent signal and $1 - \Gamma$ of the thermal radiation. The radiator is tightly integrated with an $100\,\Omega$ resistor and a RuOx sensor for local temperature control [3]. A weak thermal link between the radiator and the mixing chamber plate is constructed by a 2 mm-wide and 300 mm-long Cu braid. A dynamical balance between cooling and heating is achieved with a commercial PID temperature controller. We observe that the MXC temperature remains below 60 mK when the radiation temperature is below 1 K. A sensor mounted directly at the sample holder indicates a slightly higher sample temperature, but it remains below 80 mK for $T < 1$ K (see Fig. 3 of the main text for recorded temperatures of the two sensors).

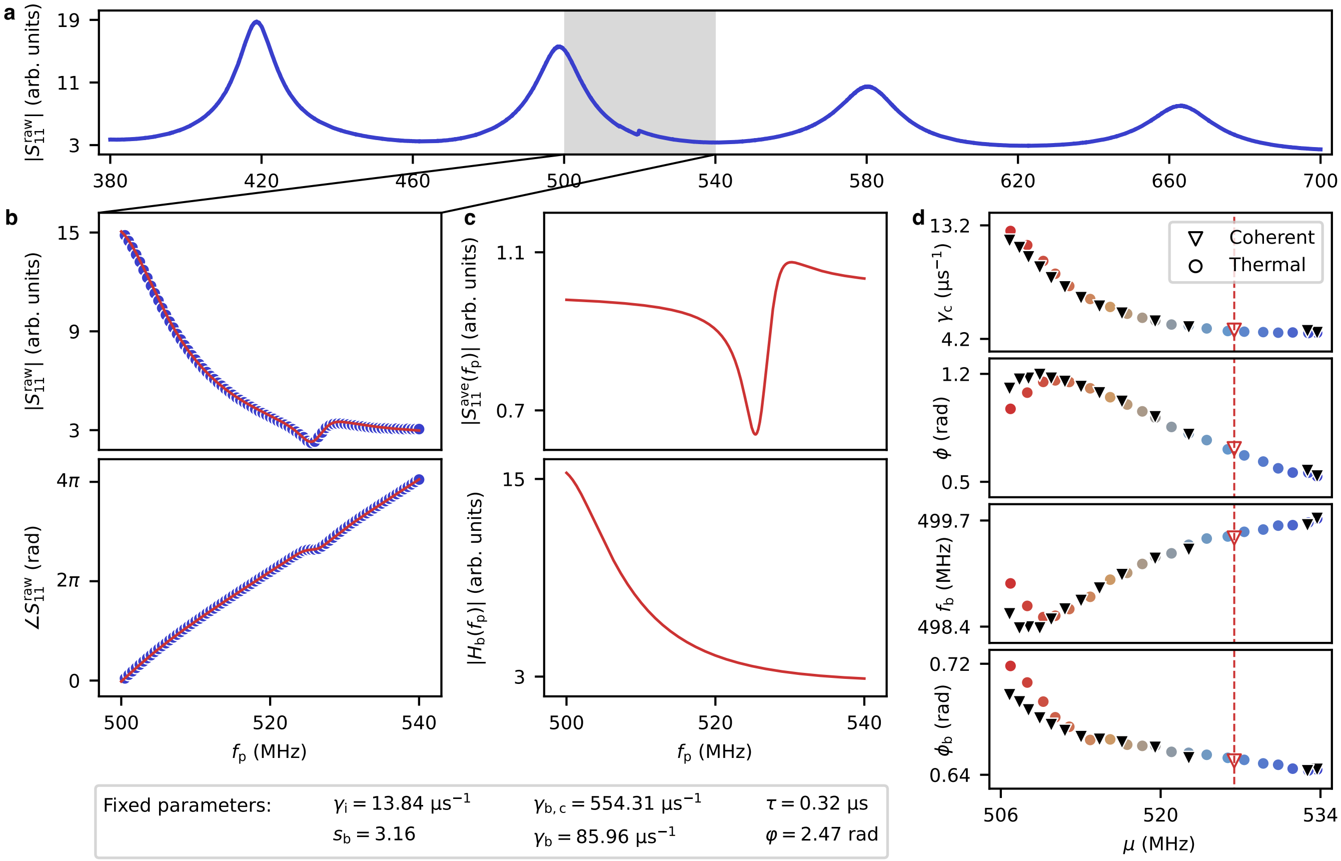

Supplementary Fig. 3: Fitting procedure for second-order correlation. **a** Magnitude of the reflection coefficients, where we observe spurious resonances with a 80 MHz spacing possibly due to a large impedance mismatch in the output path. **b** The magnitude and phase of the reflection coefficients (blue circles) together with the fitting results (red curves) in a smaller frequency range, shown as the gray area of (a). **c** The fitted ideal reflection coefficient, $S_{11}^{\rm ave}(f_{\rm p})$, and the transfer function, $H_{\rm b}(f_{\rm p})$. The legend shows the fixed parameters of the model throughout the experiment (see Methods). **d** The fitted parameters of the model for coherent (triangle) and thermal (circle) input fields. The red triangular markers are the specific values for (b) and (c).

Supplementary Note 5. DETAILED FITTING PROCEDURE

In our experiment, we observed a Lorentzian patterns in the output signal that appear at 80-MHz separations (see Supplementary Fig. 3a). This is possibly owing to a large mismatch between the sample and the HEMT amplifier (see Supplementary Fig. 2). Here, we describe the detail of the signal processing procedure that extracts the actual nanobolometer response, $S_{11}^{\rm ave}$, as well as the two parameters, μ and σ^2, from the raw data. The latter is denoted as $S_{11}^{\rm raw}$.

We phenomenologically describe the raw data as

$$S_{11}^{\rm raw}(f_{\rm p}) = e^{{\rm i}(f_{\rm p}\tau+\varphi)} H_{\rm b}(f_{\rm p}) S_{11}^{\rm ave}(f_{\rm p}), \tag{17}$$

where $H_{\rm b}(f_{\rm p}) = s_{\rm b} + {\rm e}^{{\rm i}\phi_{\rm b}}\gamma_{\rm b,c}/[(\gamma_{\rm b}/2) + i\Delta_{\rm b}]$ is the transfer function between the input and output probe signal, that describes the aforementioned mismatch induced Lorentzian pattern. Here, $s_{\rm b}$ is a scaling factor, $\Delta_{\rm b} = 2\pi(f_{\rm b} - f_{\rm p})$ is the detuning between the resonance frequency of the background Lorentzian and probe frequency, $\gamma_{\rm b,c}$ and $\gamma_{\rm b}$ are free parameters of a Lorentzian function, and $\phi_{\rm b}$ is the asymmetry factor of the lineshape. The other two parameters, τ and φ, represent the cable delay and phase offset, respectively.

In total, there are ten free parameters to be determined before extracting μ and σ^2. We first fit all the parameters at the base temperature and fix six of them throughout the experiment (see Supplementary Figs. 3b–c). The only

fitting parameters in each individual measurement are γ_c, ϕ, f_b, and ϕ_b, as shown in Supplementary Fig. 3d. The fitted results are almost identical for thermal and coherent inputs with maximally few percentage of difference at the highest temperatures ($T > 1.7\,\mathrm{K}$), which we attribute to the changing of the bath temperature T_b as measured in Fig. 3b of the main text.

In the experiment, we assume a linear relation between the thermometer frequency shift, Δf_r, and ΔT_e, as it is indicated in Fig. 2 of the main text. The photon number variance is obtained by rescaling the broadening of the reflection spectrum, i.e., $(\Delta n)^2 = \alpha^2\sigma^2$ with $\alpha = 1.97\,\mathrm{photons/MHz}$. The described signal processing procedure is used for Figs. 3 and 4 of the main text, and the Python programs can be found in the submitted source code.

[1] K. J. Blow, R. Loudon, S. J. D. Phoenix, and T. J. Shepherd, Continuum fields in quantum optics, Phys. Rev. A **42**, 4102 (1990).

[2] N. B. Grosse, T. Symul, M. Stobińska, T. C. Ralph, and P. K. Lam, Measuring photon antibunching from continuous variable sideband squeezing, Phys. Rev. Lett. **98**, 153603 (2007).

[3] J. Goetz, S. Pogorzalek, F. Deppe, K. G. Fedorov, P. Eder, M. Fischer, F. Wulschner, E. Xie, A. Marx, and R. Gross, Photon statistics of propagating thermal microwaves, Phys. Rev. Lett. **118**, 103602 (2017).

[4] D. M. Pozar, *Microwave Engineering*, 4th ed. (Wiley, Hoboken, 2011).

[5] Q.-M. Chen, M. Partanen, F. Fesquet, K. E. Honasoge, F. Kronowetter, Y. Nojiri, M. Renger, K. G. Fedorov, A. Marx, F. Deppe, and R. Gross, Scattering coefficients of superconducting microwave resonators. II. System-bath approach, Phys. Rev. B **106**, 214506 (2022).

[6] W. Demtröder, *Laser Spectroscopy*, 2nd ed. (Springer, Heidelberg, 1996).
