# Supplementary figures and images for: Correlation measurement of propagating microwave photons at millikelvin
Source: Nat Commun. 2025 Apr 24;16:3875. doi: 10.1038/s41467-025-59230-2 (PMC12022262; doi:10.1038/s41467-025-59230-2)

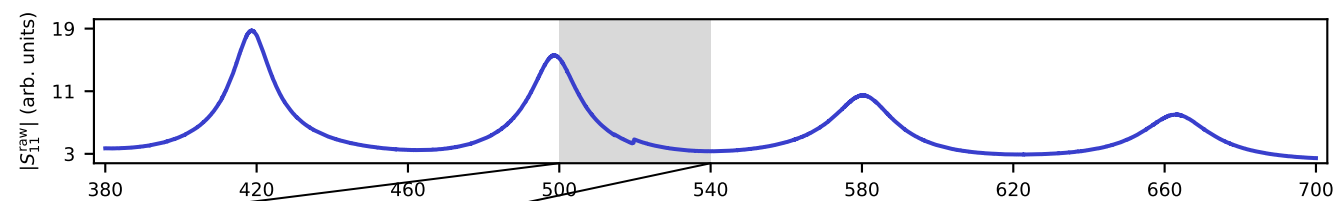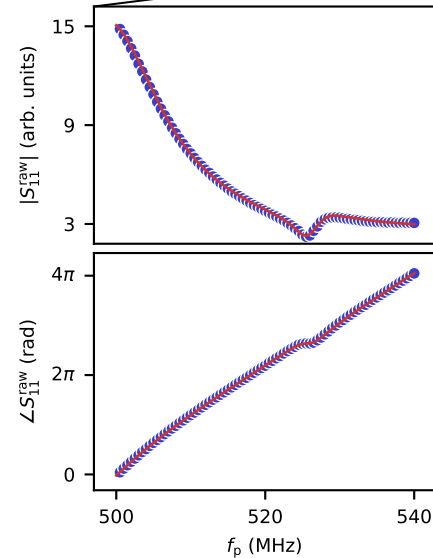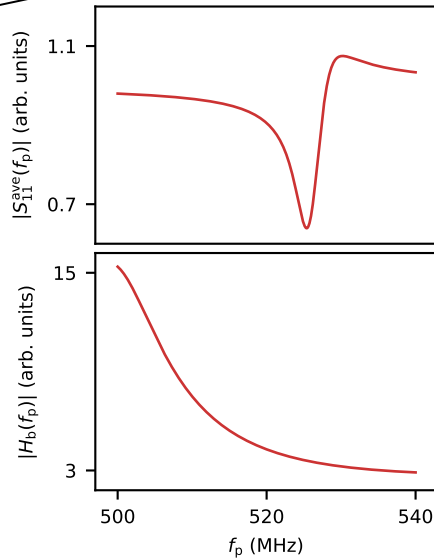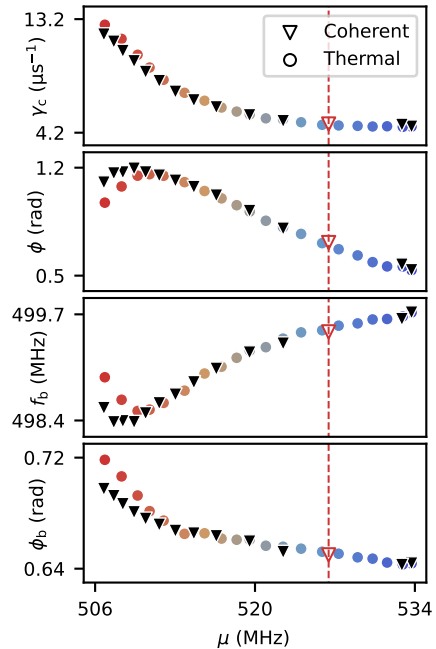

Fixed parameters:  $\gamma_i = 13.84 \mu\text{s}^{-1}$   $\gamma_{b,c} = 554.31 \mu\text{s}^{-1}$   $\tau = 0.32 \mu\text{s}$   
 $s_b = 3.16$   $\gamma_b = 85.96 \mu\text{s}^{-1}$   $\phi = 2.47 \text{ rad}$

Supplement: Supplementary file 3 — Source Data [file 41467_2025_59230_MOESM3_ESM.zip › Source_Data/output/bolometer_g2_ext_fig.pdf]

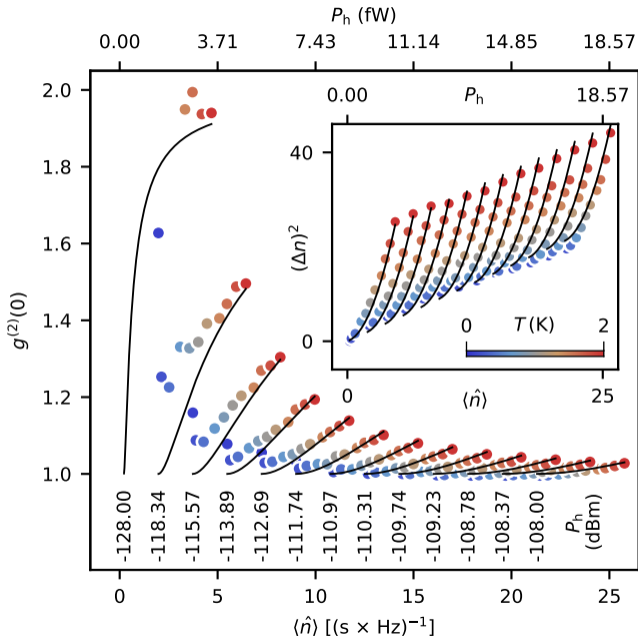

Supplement: Supplementary file 3 — Source Data [file 41467_2025_59230_MOESM3_ESM.zip › Source_Data/output/bolometer_g2_fig4.pdf]

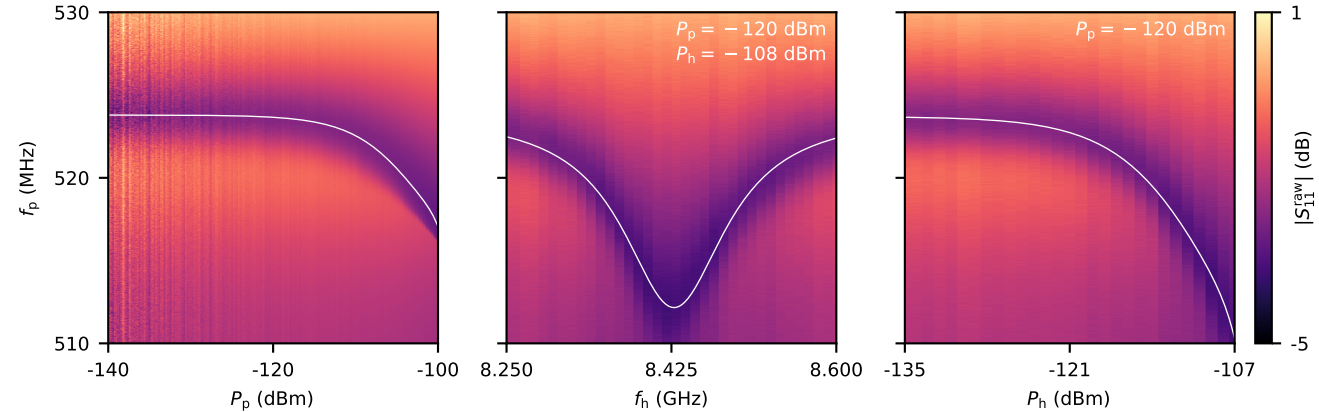

Supplement: Supplementary file 3 — Source Data [file 41467_2025_59230_MOESM3_ESM.zip › Source_Data/output/bolometer_g2_fig1.pdf]

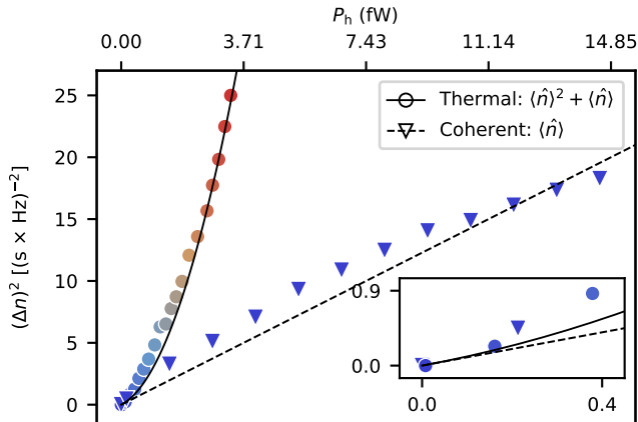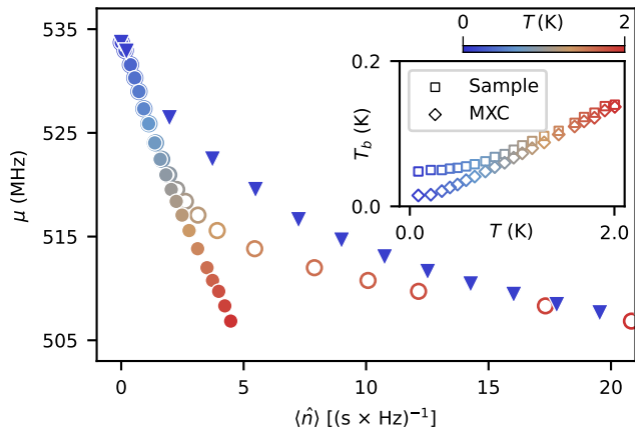

Supplement: Supplementary file 3 — Source Data [file 41467_2025_59230_MOESM3_ESM.zip › Source_Data/output/bolometer_g2_fig3.pdf]

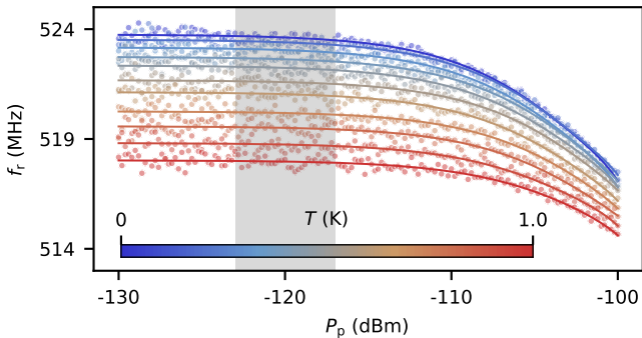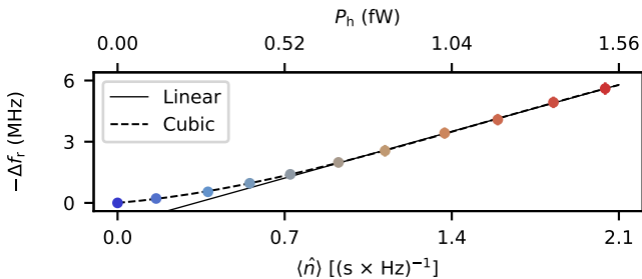

Supplement: Supplementary file 3 — Source Data [file 41467_2025_59230_MOESM3_ESM.zip › Source_Data/output/bolometer_g2_fig2.pdf]
